# Combining Pembrolizumab and Ofatumumab in Triple-Negative Breast Cancer and Multiple Sclerosis: A Case Report

**DOI:** 10.70352/scrj.cr.26-0383

**Published:** 2026-09-10

**Authors:** Haruna Iriyama, Madoka Iwase, Yuko Takano, Dai Takeuchi, Takahiro Ichikawa, Reiko Ohata, Yuri Ozaki, Misato Yamamoto, Gai Inaguma, Nao Torii, Chihiro Toyoda, Kana Kawabe, Toyone Kikumori, Norikazu Masuda

**Affiliations:** 1 Department of Breast and Endocrine Surgery, Nagoya University Hospital, Nagoya, Aichi, Japan; 2 Department of Breast Surgery, Graduate School of Medicine, Kyoto University, Kyoto, Kyoto, Japan

**Keywords:** triple-negative breast cancer, pembrolizumab, multiple sclerosis, ofatumumab

## Abstract

**INTRODUCTION:**

Pembrolizumab has been widely used since its approval as perioperative therapy for triple-negative breast cancer. While the emergence of diverse antibody drugs has expanded therapeutic options in recent years, reports on the combined use of immune-related antibodies in patients with other diseases remain limited.

**CASE PRESENTATION:**

A 42-year-old woman presented with a 5-cm mass in her right breast. Needle biopsy confirmed invasive ductal carcinoma (cT2N0M0, cStage IIB, triple-negative breast cancer). Pembrolizumab-based therapy was considered, but the patient was already receiving ofatumumab, an anti-CD20 antibody, for multiple sclerosis (MS). After consultation with a neurologist, she underwent neoadjuvant chemotherapy with paclitaxel, carboplatin, and pembrolizumab, followed by epirubicin, cyclophosphamide, and pembrolizumab. Preoperative imaging indicated a clinical complete response. She underwent right partial mastectomy with sentinel lymph node biopsy, and postoperative pathology confirmed a pathologic complete response. No exacerbation of MS has been observed to date.

**CONCLUSIONS:**

This case demonstrates that pembrolizumab can be safely administered for triple-negative breast cancer in a patient receiving ofatumumab for MS, achieving a pathologic complete response.

## Abbreviations

DMT
: disease-modifying therapy
ER
: estrogen receptor
HER 2
: human epidermal growth factor receptor 2
ICI
: immune checkpoint inhibitor
irAE
: immune-related adverse event
IS
: intensity score
MS
: multiple sclerosis
PgR
: progesterone receptor
PS
: proportion score
TS
: total score
UICC
: Union for International Cancer Control

## INTRODUCTION

Pembrolizumab has been widely used since its approval by Japanese Insurance in 2022 as perioperative therapy for triple-negative breast cancer, and opportunities to administer it to patients with underlying diseases have increased. Although the development of various antibody drugs has expanded treatment options, reports on the combined use of immune-related antibodies in patients with other diseases remain scarce. Ofatumumab, an anti-CD20 antibody that depletes B cells from the circulation, is approved for relapse prevention and disease control in MS, an autoimmune disease. We report a case in which pembrolizumab was safely and successfully administered together with ofatumumab.

## CASE PRESENTATION

A 42-year-old woman presented to a previous clinic with a rapidly enlarging mass in her right breast. A biopsy confirmed breast cancer, and she was referred to Nagoya University hospital for treatment.

Her past medical history included MS, diagnosed at the age of 20, and an ovarian dermoid tumor under observation. She began subcutaneous injections of ofatumumab around the age of 40 and continued the treatment for 2 years and 3 months. There was no family history of malignancy.

**Fig. 1:**
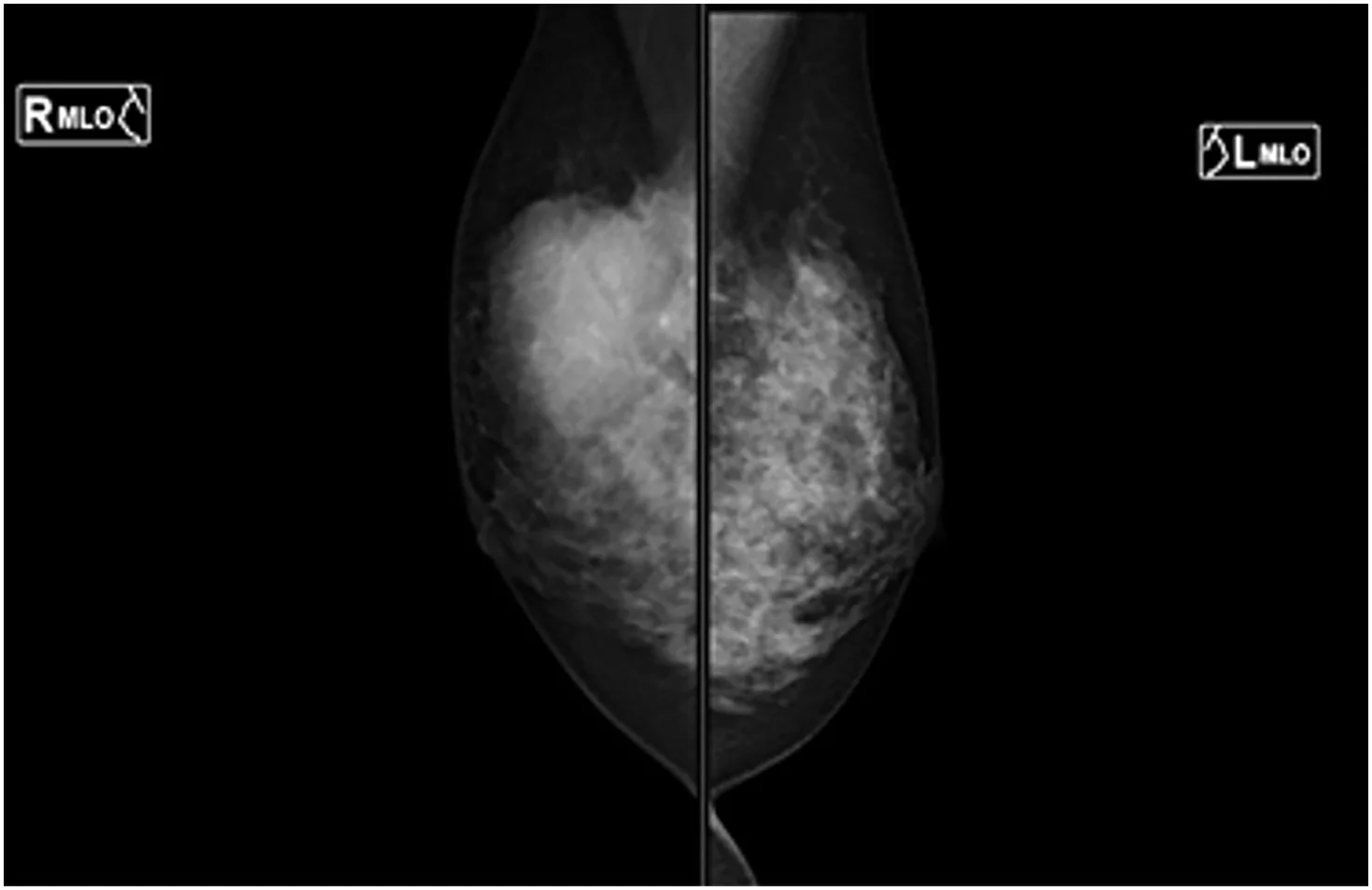
Mammography findings (MLO view). A high-density lobulated mass was observed in the right upper–outer region. MLO, mediolateral oblique

**Fig. 2:**
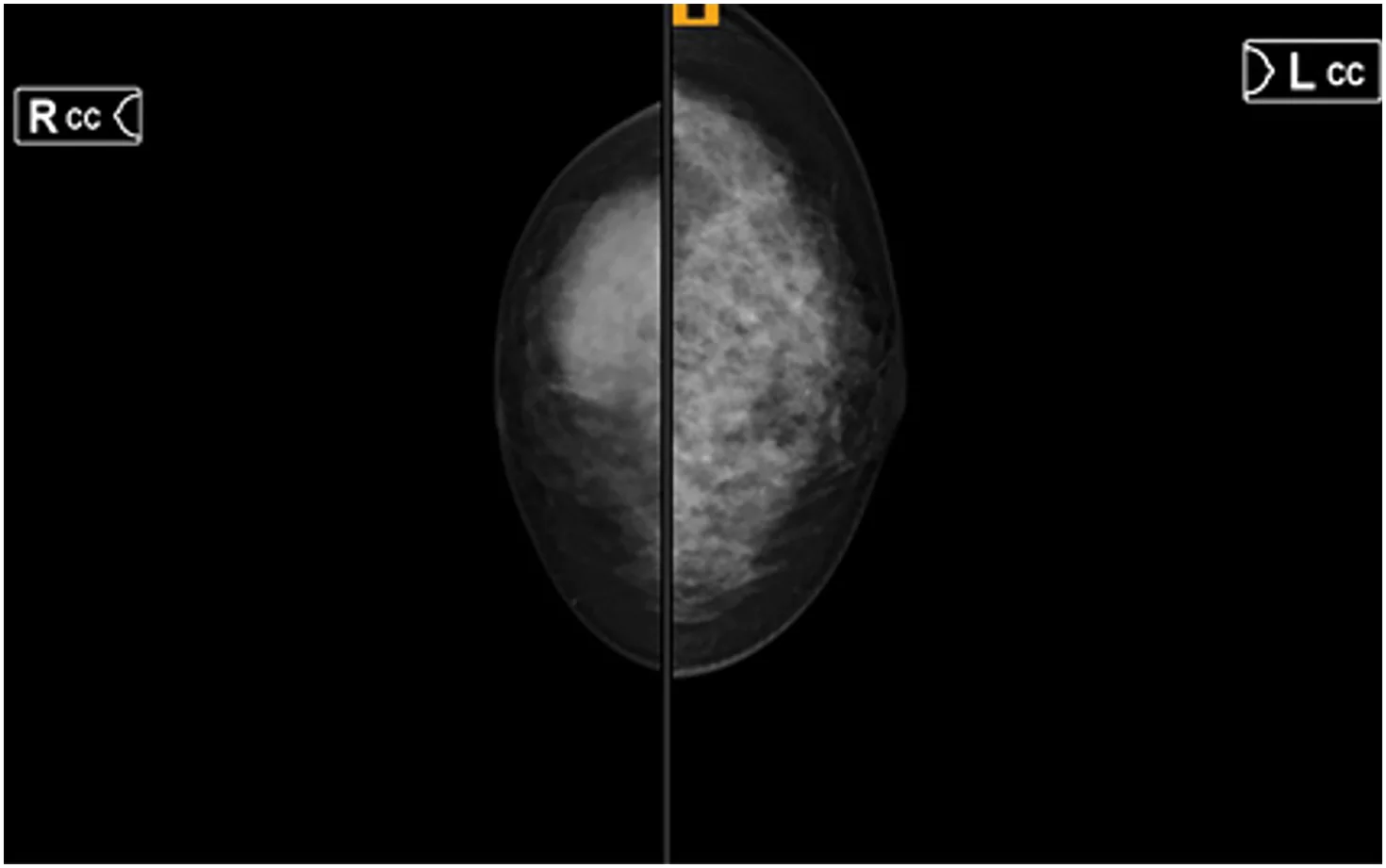
Mammography findings (CC view). CC, craniocaudal

**Fig. 3:**
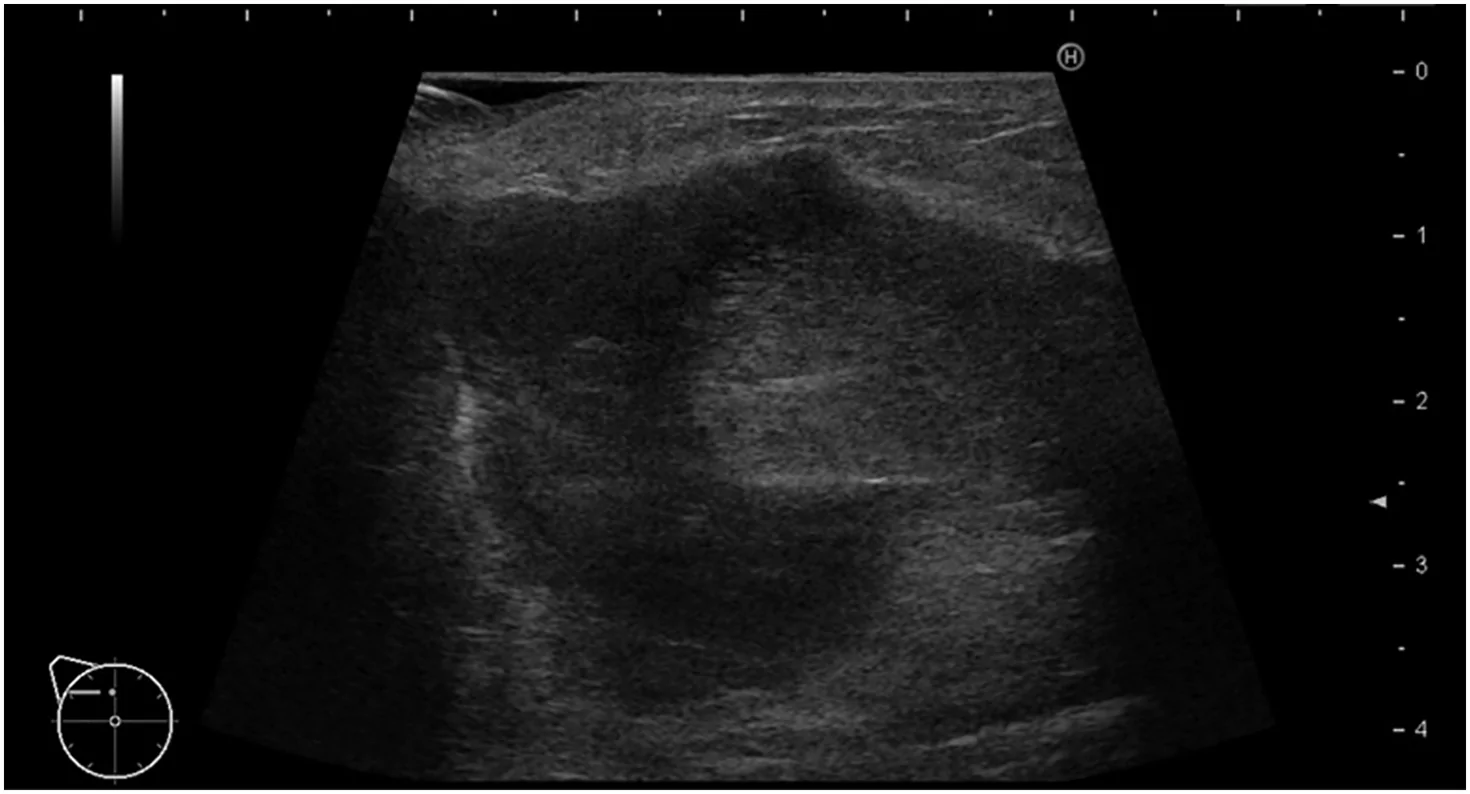
Breast ultrasonography findings. A lobulated mass measuring 40 × 30 × 50 mm was detected in the right breast. The mass contained both cystic and solid components, and intratumoral hemorrhage was suspected.

**Fig. 4:**
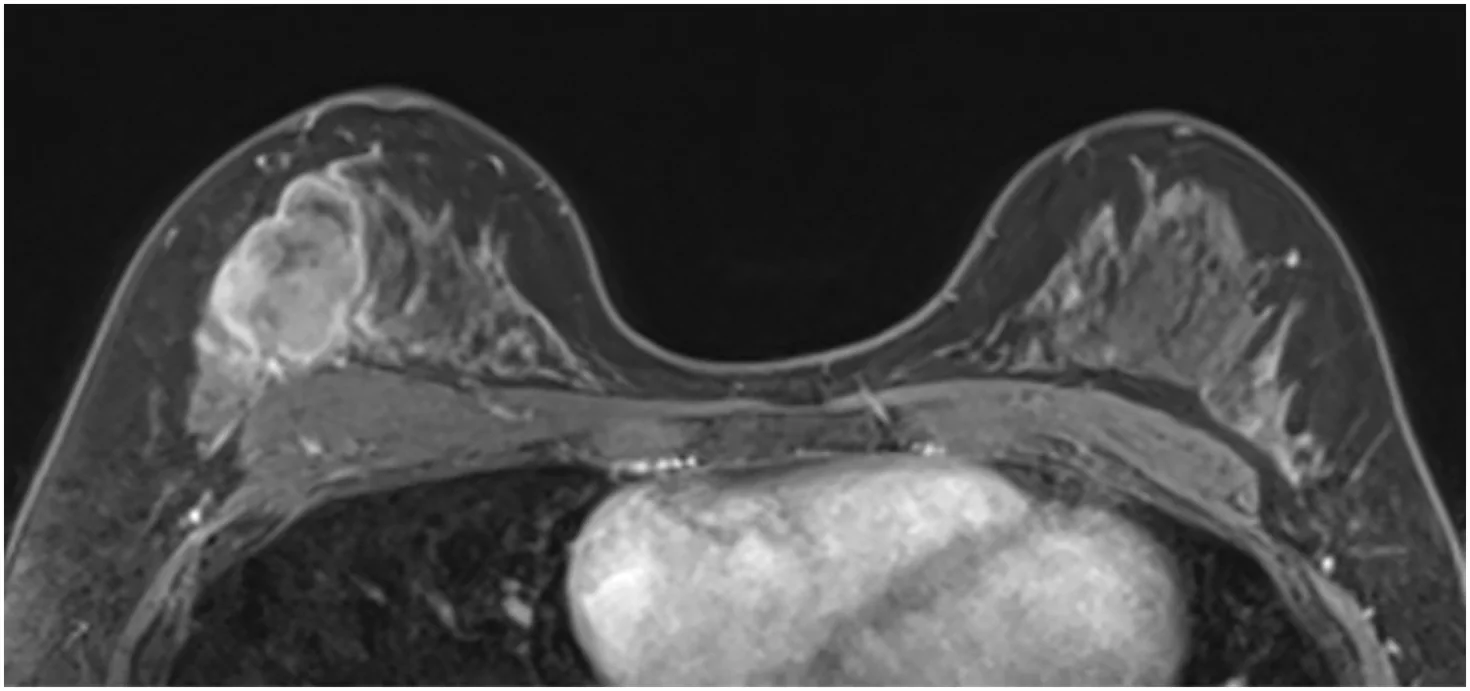
Contrast-enhanced MRI findings (before initiation of neoadjuvant chemotherapy). A solid lobulated mass measuring 38 mm with ring enhancement was observed in the right breast.

On physical examination, a mass measuring approximately 5 cm was noted in the right breast. Mammography revealed a high-density lobulated mass in the right Upper-Outer region (**Figs. 1** and **2**). Breast ultrasonography demonstrated a lobulated mass measuring 40 × 30 × 50 mm in the right breast, with both cystic and solid components, suggesting intratumoral hemorrhage (**Fig. 3**). Contrast-enhanced MRI showed a solid lobulated mass with ring enhancement, measuring 38 mm in diameter (**Fig. 4**). CT revealed a 34-mm mass in the same region, with no evidence of distant metastasis.

Core needle biopsy confirmed invasive ductal carcinoma, histological grade 3, with ER score: PS 0 + IS 0 = TS 0 (Allred score), PgR score: PS 0 + IS 0 = TS 0, HER2 score 0, and a Ki-67 index of 20%–30%. Genetic testing for *BRCA1/2* mutations was negative.

Based on these findings, the patient was diagnosed with right breast cancer, cT2N0M0, Stage IIA (UICC), triple-negative breast cancer.

Because the patient had high-risk triple-negative breast cancer, neoadjuvant chemotherapy with pembrolizumab according to the KEYNOTE-522 regimen was considered. As she was undergoing treatment for MS, an autoimmune disease, the safety of pembrolizumab was a concern. After a multidisciplinary conference with neurologists, we decided to continue ofatumumab with regular follow-up while administering pembrolizumab.

Treatment was initiated with paclitaxel (80 mg/m^2^), carboplatin (AUC 1.5), and pembrolizumab (200 mg). Dexamethasone sodium phosphate 6.6 mg was administered as an antiemetic at each cycle of chemotherapy. On day 7 of cycle 3, she developed thyrotoxicosis due to destructive thyroiditis and was started on bisoprolol fumarate. By day 20 of cycle 4, she developed hypothyroidism and was started on levothyroxine sodium, after which pembrolizumab could be continued without interruption. Breast ultrasonography showed tumor shrinkage to approximately 20 × 10 × 20 mm.

Subsequently, therapy with epirubicin (90 mg/m^2^), cyclophosphamide (600 mg/m^2^), and pembrolizumab (200 mg) was initiated. Dexamethasone sodium phosphate 9.9 mg was administered as an antiemetic at each cycle of chemotherapy. On day 17 of cycle 1, she developed febrile neutropenia. On day 12 of cycle 3, she received ofatumumab and developed fever, but brain MRI showed no worsening of MS. The regimen was completed through 4 cycles without further significant adverse events.

We did not synchronize the administration of ofatumumab with chemotherapy. Ofatumumab was given 10 days before the initiation of chemotherapy, followed by self-injections every 4 weeks.

**Fig. 5:**
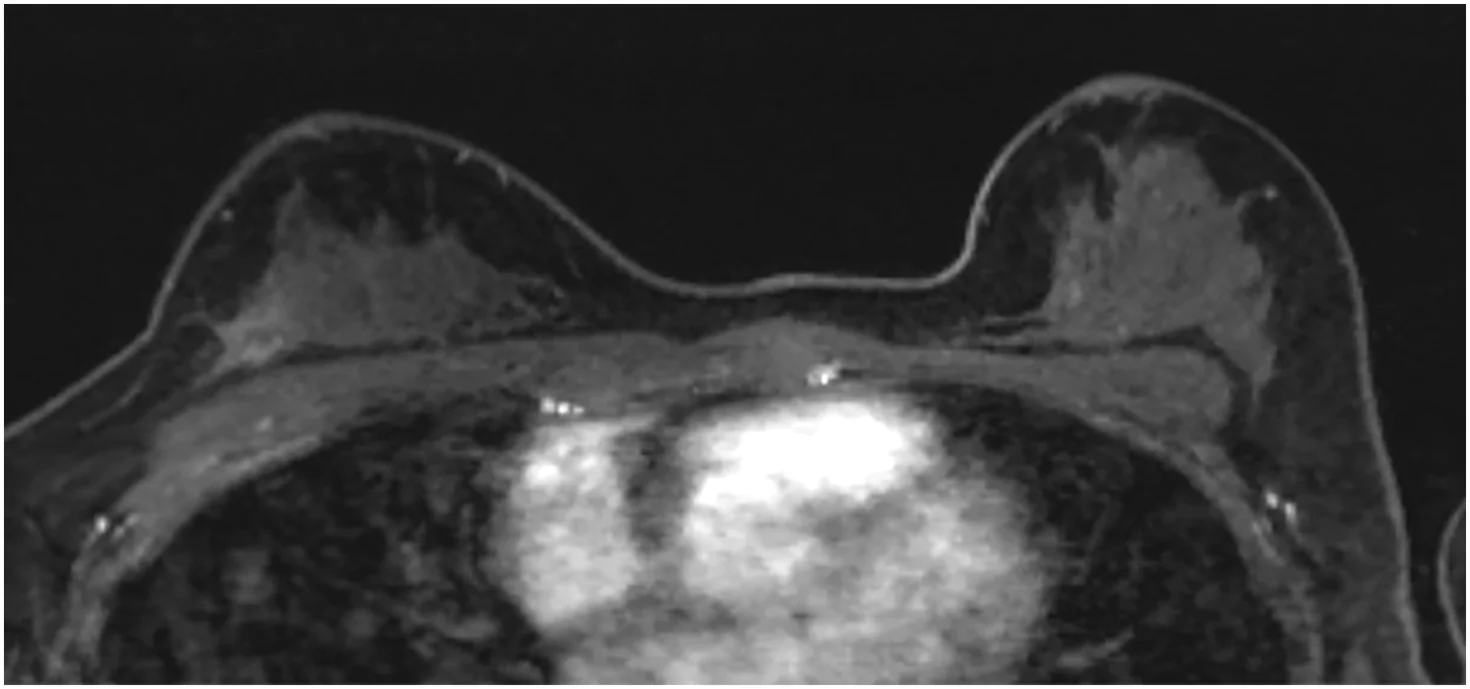
Contrast-enhanced MRI findings (after completion of neoadjuvant chemotherapy). Marked tumor shrinkage was noted, and no enhancement was observed.

Post-treatment contrast-enhanced MRI revealed marked tumor shrinkage (**Fig. 5**), and a clinical complete response was judged. The patient underwent right breast partial resection with sentinel lymph node biopsy. Sentinel nodes were negative (0/3), and axillary dissection was omitted.

Histopathological examination after surgery showed no residual tumor, ypT0, ypN0, with no lymphovascular invasion and negative margins, confirming a pathological complete response, a treatment effect grade of 3, based on the 19th edition of the Japanese Breast Cancer Society Breast Cancer Guidelines.

As postoperative therapy, hypofractionated irradiation of the residual breast (43.2 Gy/16 fractions) was performed, followed by 9 courses of pembrolizumab (200 mg). Brain MRI after completion of pembrolizumab showed no evidence of MS progression, and perioperative therapy was successfully completed. And at present, there is no evidence of recurrence of either breast cancer or MS.

## DISCUSSION

In our case, we achieved favorable outcomes by administering an ICI to a patient with triple-negative breast cancer and pre-existing MS, while maintaining DMT. Notably, the patient’s MS remained clinically and radiologically stable throughout treatment.

Pembrolizumab is a humanized anti–PD-1 monoclonal antibody that exerts antitumor effects by reactivating T-cell immunity.^1)^ In breast cancer, it is approved for perioperative therapy in high-risk triple-negative disease and for unresectable or recurrent PD-L1–positive triple-negative breast cancer, and its use has become widespread. However, careful management of irAEs is required, and patients with autoimmune disease who had received systemic therapy within 2 years were excluded from the KEYNOTE-522 trial.^2)^ Therefore, patients with coexisting autoimmune diseases or a history of chronic or recurrent autoimmune disorders require careful administration because irAEs may occur or worsen.

Meanwhile, MS in our case is a demyelinating disease of the central nervous system, and depending on the site of involvement, it can cause a wide range of symptoms. MS is a relatively uncommon disease, with an estimated prevalence of approximately 14 per 100000 in Japan. MS typically presents in young adults and follows a chronic course characterized by relapses and remissions, with some patients experiencing gradual progression.^3)^ Its pathogenesis is thought to be autoimmune, involving B-cell antibodies and activated T cells that contribute to myelin injury.^4)^

High-dose intravenous corticosteroids remain the first-line treatment for acute MS relapses, with plasma exchange reserved for insufficient responders. Early initiation of DMT after diagnosis is recommended to reduce relapse frequency and prevent the progression of physical disability.^3)^

Ofatumumab, one of the DMTs, is an anti-CD20 monoclonal antibody that depletes circulating B cells to suppress demyelination and is used to prevent relapse and slow the progression of MS. In 2021, it was approved in Japan as the first B-cell–targeted therapy for MS.^5)^

As a general approach to monitoring MS, it is recommended to perform brain MRI annually during clinically stable periods and additionally when relapse is suspected.^3)^

In our patient, concerns existed that pembrolizumab might worsen MS by enhancing T-cell activity, or that ofatumumab might reduce the antitumor effect of pembrolizumab by suppressing T-cell activation. After consultation with neurologists, ofatumumab was continued and pembrolizumab was administered, resulting in completion of the planned therapy and achievement of a pathological complete response. Brain MRI performed during an episode of fever in the course of chemotherapy, as well as after completion of breast cancer therapy, showed no signs of MS exacerbation.

We searched the Japan Medical Abstracts Society database and PubMed using the keywords “pembrolizumab” and “multiple sclerosis,” as well as “ICI” and “multiple sclerosis,” supplemented by manual hand-searching.

We found no reports in Japan on the use of ICIs, including pembrolizumab, in patients with MS, although a few studies have been published in other countries. While several cohort studies and meta-analyses^6–10)^ have examined MS relapse associated with ICI use (**Table 1**), to our knowledge, no previous report has specifically described ICI administration during ofatumumab therapy.

**Table 1:** Summary of published reports on ICI therapy in patients with MS

First author (year)	Study patients	Sample size, N	Cancer type, N (%)	MS activity at ICI initiation, N (%)	Patients continuing DMT, N (%)	Types of DMT	Clinical relapse of MS, N (%)	Relapsed patients continuing DMT, N	Relapsed patients discontinuing DMT, N	Treatment efficacy for cancer	Key findings	Comments
Fletcher^6)^ (2025)	Patients with a history of MS	45	NR	NR	NR	NR	8 (18%)	2	6	NR	MS worsening during ICI treatment: 18%	
Hirunpattarasilp^7)^ (2025)	Patients with a history of MS	12	Lung 8, breast 2, other 2	RRMS 11 (92%), unknown 1 (7%)	4 (100%), 8 patients were not receiving DMT at baseline.	DMF2, GA1, IFN-β 1	0 (0%)	–	–	CR, PR 6 (50%)	No patients experienced MS worsening during ICI treatment.	
Gelibter^8)^ (2025)	Patients with a history of MS	90	Melanoma (45%), lung (33.3%), other (21.7%)	NR	NR	NR	3 (3%)	NR	NR	NR	The MS relapse rate during ICI treatment was 5.45 per 100 patient years.	Meta-analysis including studies by Chavaz, Conway, Androdias, Nylander, Quinn.
Afzal^9)^ (2025)	Patients with a history of MS	27	Lung 12, melanoma 5, genitourinary 5, other 5	RRMS 19 (73%), PPMS 2 (9%), SPMS 1 (4%), other 5 (19%)	4 (36%), 16 patients were not receiving DMT at baseline.	OCR3, GA 3, FIN 2, NTZ 2, DMF1	1 (4%)	–	1	NR	MS worsening during ICI treatment: 4%	This study compared relapse rates between MS patients treated with ICI and those not treated with ICI, and found no increase in relapse associated with ICI use.
Garcia^10)^ (2019)	Patients with MS relapse or newly diagnosed MS	NR	Melanoma 7, lung 2, other 3, unreported 2	NR	1 (25%), 4 patients were not receiving DMT at baseline.	GA 3, IFN-β 1	Relapse: 8 patients, New onset: 6	4	4	NR	MS relapse is uncommon, but fatal cases have been reported.	This study used the FAERS database to identify patients who were diagnosed with MS or experienced MS relapse during ICI therapy; however, the total number of patients with preexisting MS is unknown.

This table summarizes cohort studies and meta-analyses on MS relapse during ICI therapy. To our knowledge, no previous report has specifically described ICI administration during ofatumumab therapy. Cancer types with few cases were grouped under “Other.”

DMF, dimethyl fumarate; DMT, disease-modifying therapy; FIN, fingolimod; GA, glatiramer acetate; ICI, immune checkpoint inhibitor; IFN-β, interferon beta; MS, multiple sclerosis; NR, not reported; NTZ, natalizumab; OCR, ocrelizumab; PPMS, Primary progressive MS; RRMS, relapsing-remitting MS; SPMS, Secondary progressive MS; CR, complete response; PR, partial response; FAERS, US Food and Drug Administration Adverse Event Reporting System

According to Gelibter et al.,^8)^ a meta-analysis of 90 patients with a history of MS showed that the relapse rate with clinical symptoms after ICI administration was 0.0545 per person-year, and the rate of new MRI findings was 0.249 per person-year, indicating that the relapse risk is not high. They also identified discontinuation of DMT for MS as a relapse risk factor: 60% of patients who relapsed were not receiving DMT at the start of ICI therapy, 80% of those with new MRI findings had stopped DMT when ICI was initiated, and 40% had discontinued natalizumab, a drug known to cause rebound after withdrawal. They further noted that MS relapses often occur within 6 months of starting ICI treatment, suggesting that careful follow-up after initiation may allow ICIs to be used safely in patients with MS. Furthermore, Conway et al.^11)^ reported that factors associated with an increased risk of MS disease activity include younger age (<50 years), a high relapse rate or high radiographic disease activity prior to the initiation of ICIs, and discontinuation of anti-trafficking agents such as natalizumab or sphingosine-1-phosphate receptor modulators. Conversely, they noted that older age (>50 years), a low relapse rate or low radiographic activity before starting ICI therapy, concurrent use of cytotoxic chemotherapy, and concomitant use of DMTs may decrease the risk of disease activity.

Previous studies^8,11)^ have suggested that discontinuing DMT—particularly natalizumab—may increase the risk of MS relapse, but no cases were found in which a patient receiving ofatumumab for MS discontinued it before starting ICI therapy.

There is a report of an MS patient receiving ocrelizumab (an anti-CD20 antibody not approved for MS in Japan) who discontinued it after being diagnosed with stage IIA breast cancer and was treated with a pembrolizumab-containing regimen; although new demyelinating lesions appeared on MRI, the patient remained asymptomatic.^12)^ Similarly, another report described an MS patient on ocrelizumab who discontinued it after being diagnosed with sarcomatoid renal cell carcinoma and received a pembrolizumab-containing regimen without MS relapse.^13)^ No severe outcomes were reported as a result of stopping ocrelizumab at the time of cancer diagnosis, but because the number of cases is small, it remains unclear whether ocrelizumab or ofatumumab should be discontinued when malignancy is diagnosed. At the very least, for patients whose MS is stable on a DMT, including ofatumumab, it may be preferable to administer ICIs without discontinuing DMT.

On the other hand, Garcia et al.^10)^ analyzed cases from the FDA database, published case reports,^14–16)^ and their own institution, and identified 14 patients who developed or relapsed MS during a 2-year period while receiving pembrolizumab, atezolizumab, nivolumab, ipilimumab, avelumab, or durvalumab. Eight of these patients had a prior history of MS, 1 experienced MS worsening, and 2 died. Because the total number of treated patients was not reported, the relapse rate could not be determined. However, the presence of severe outcomes^10)^ suggests that careful monitoring is required when administering ICIs.

In the present case, the patient experienced no relapse of MS during the course of treatment. However, reports of restarting ICI therapy after an MS relapse during ICI administration are scarce, with only a single case described by Iglesias Martinez et al.^17)^

Moreover, when patients without a history of MS develop MS as an irAE during ICI therapy, there are no clear data on whether ICIs can be safely resumed under concomitant DMT, and at present, resuming ICIs cannot be recommended.

Although the number of reported cases remains limited, several studies have described the safety of ICIs in patients with MS. However, only a few reports have evaluated the therapeutic efficacy of ICIs in the context of concurrent DMT. According to the report by Hirunpattarasilp et al.,^7)^ among 12 patients with a history of MS who received ICIs as anticancer therapy, the initial assessment after a median of 7 ICI cycles showed complete response in 1 patient (8.3%), partial response in 5 patients (41.7%), stable disease in 4 patients (33.3%), and progressive disease in 2 patients (16.7%), resulting in an objective response rate of 50%. Furthermore, 4 of the 12 patients maintained complete or partial responses for more than 6 months, yielding a durable response rate of 33.3%. Notably, overall survival and progression-free survival did not differ between patients with and without DMT use. These findings suggest that concomitant DMT may not substantially affect the therapeutic efficacy of ICIs.

Despite these observations, the number of reported cases remains small, and further accumulation of clinical data is needed. Because treatment selection was challenging at the start of therapy in our case, we believe that reporting this case has significant value.

## CONCLUSIONS

This case represents the first report of combined therapy with the anti-CD20 antibody ofatumumab and pembrolizumab. Pembrolizumab was administered while continuing ofatumumab for MS, and no relapse of MS was observed. Neoadjuvant chemotherapy including pembrolizumab achieved a favorable therapeutic outcome. Because evidence regarding the risk of MS relapse and the impact on antitumor efficacy remains limited, careful administration under specialist follow-up is recommended.

## DECLARATIONS

### Use of artificial intelligence tools

The authors used Copilot to refine the language and improve the readability of the manuscript. The authors carefully reviewed and edited the output and take full responsibility for the final version of the manuscript.

### Availability of data and materials

The data used in the current study are available from the corresponding author on reasonable request.
